# Frequency of antiseptic resistance genes in clinical staphycocci and enterococci isolates in Turkey

**DOI:** 10.1186/s13756-017-0244-6

**Published:** 2017-08-30

**Authors:** Seyda Ignak, Yasar Nakipoglu, Bulent Gurler

**Affiliations:** 1 Department of Medical Biology, Bahcesehir University School of Medicine, Sahrayıcedid Mah. Batman Sok. No:66-68, Yenisahra-Göztepe, Istanbul, Turkey; 2 Istanbul Faculty of Medicine, Department of Medical Microbiology, Istanbul University, Istanbul, Turkey

**Keywords:** *Staphylococcus spp.*, *Enterococcus spp.*, *qac* genes, Antiseptic, Biocide, Resistance

## Abstract

**Background:**

Disinfectants and antiseptics are biocides widely used in hospitals to prevent spread of pathogens. It has been reported that antiseptic resistance genes, *qac*’s, caused tolerance to a variety of biocidal agents, such as benzalkonium chloride (BAC) and chlorhexidine digluconate (CHDG) in *Staphylococcus spp*. isolates. We aimed to search the frequency of antiseptic resistance genes in clinical *Staphylococcus spp.* and *Enterococcus spp*. isolates to investigate the possible association with antiseptic tolerance and antibiotic resistance.

**Methods:**

Antiseptic resistance genes (*qacA/B*, *smr*, *qacG*, *qacH*, and *qacJ)* isolated from Gram-positive cocci (69 *Staphylococcus spp*. and 69 *Enterococcus spp*.) were analyzed by PCR method. The minimum inhibitory concentrations (MICs) of BAC and CHDG were determined by agar dilution method, whereas antibiotic susceptibility was analyzed by disk diffusion method according to Clinical and Laboratory Standards Institute (CLSI) criteria.

**Results:**

The frequency of antiseptic resistance genes was found to be high (49/69; 71.0%) in our clinical staphylococci isolates but absent (0/69; 0%) in enterococci isolates. The frequency of *qacA/B* and *smr* genes was higher (25/40; 62.5% and 7/40; 17.5%, respectively) in coagulase negative staphylococci (CNS) when compared to *Staphylococcus aureus* strains (3/29; 10.3%, and 4/29; 13.8%, respectively). In contrast, the frequency of *qacG* and *qacJ* genes was higher (11/29; 37.9% and 8/29; 27.5%, respectively) in *S. aureus* than those of CNS (5/40; 12.5%, 10/40; 25.0%) strains. *qacH* was not identified in none of the strains. We found an association between presence of antiseptic resistance genes and increased MIC values of BAC (>4 μg/mL) in staphylococci and it was found to be statistically statistically significant (*p* < 0.01). We also showed that MICs of BAC and CHDG of vancomycin-resistant enterococci (VRE) isolates were significantly higher than those of vancomycin-susceptible enterococci (VSE) isolates (*p* < 0.01).

**Conclusions:**

For our knowledge, our study is the first to investigate antiseptic resistance genes in enterococci and also *qacG*, *qacH*, and *qacJ* genes in staphylococci isolates in Turkey. Further studies are needed to revise the biocide policy and to support infection control programs to avoid the development of new resistance mechanisms.

## Background

*Staphylococcus aureus* causes a wide range of clinical infections such as skin and soft tissue infections, surgical site infections, endocarditis, and bacteremia. Traditionally, coagulase-negative staphylococci (CNS) isolates were considered a as non-pathogenic commensals but in recent years the clinical importance of CNS has been increased significantly due to its role in some diseases like catheter related bloodstream infections. *Enterococcus spp.* found in the gastrointestinal tract of humans is a commensal bacteria and considered to be harmless for many years. Nowadays, it is considered to be one of the most common nosocomial pathogen especially due to strains of vancomycin resistant enterococci, VRE [1, 2]. There has been a significant increase in the incidence of nosocomial infections caused by *Staphylococcus spp*. and *Enterococcus spp*. which are multiresistant to various antibiotics in recent years [3].

Some antiseptic and disinfectant agents in the hospital setting such as quaternary ammonium compounds like benzalkonium chloride (BAC) and divalent cations like chlorhexidine digluconate (CHDG) are used to prevent infections within healthcare facilities. It has been reported in many studies that extensive use of biocidal agents reduce susceptibility to antiseptics in *Staphylococcus spp*. [4–6].

Antiseptic resistance genes (*qacAB*, *smr*, *qacG*, *qacH*, *qacJ*), encoding multidrug efflux pumps which are carried by plasmids, were identified in *Staphylococcus* genus for the first time [7–10]. Antiseptic resistance gene studies of *qacA/B* and *smr* mostly focused on *S. aureus* [10–12] but there are a limited number of studies in CNS strains [13, 14]. Moreover, the studies concerning the prevalance of *qacG*, *qacH*, and *qacJ* genes mostly focused on CNS obtained from animal and food sources [15, 16].

Despite the rising significance of *Enterococcus spp.* as nosocomial pathogen there are insufficient number of studies on *qac* genes in clinical isolates. Bischoff et al. [17] detected the first *qacA/B* gene-positive *Enterococcus faecalis* isolate in clinical blood samples. Thus far, there are no *qac* genes reported in enterococci other than *qacC*, *qacEΔ1, qacZ* and *qacA/B*. Increase in minimum inhibitory concentration (MIC) values to biocides does not mean “resistance” because these agents can be used at high concentrations without encountering toxicity [18]. Thus the terms “reduced susceptibility” or “increased tolerance” are more suitable for pathogens exhibiting an elevated MIC to a biocides [19].

In this study, we aimed; 1. To investigate *qacAB*, *smr*, *qacG*, *qacH*, *qacJ* genes in *Staphylococcus spp*. and *Enterococcus spp*. isolates, 2. To determine the susceptibility of the strains to antibiotics and two antiseptic agents, including BAC and CHDG, 3. To show relationship between MIC values of antiseptic agents/resistance to antibiotics and presence of antiseptic resistance genes. For our knowledge, this is the first study in Turkey that investigated antiseptic resistance genes in enterococci isolates and also first in terms of showing the presence of *qacH, qacG,* and *qacJ* genes in staphylococci isolates.

## Methods

## Bacterial isolates

In our study, 69 *Staphylococcus spp*. (10 methicillin-resistant *S.aureus* (MRSA), 19 methicillin-susceptible *S.aureus* (MSSA), 27 MRCNS, 13 of methicillin-susceptible CNS (MSCNS)) and 69 of *Enterococcus spp*. were collected from different clinical samples such as blood, cerebrospinal fluid, urine, abscess, catheter tips, nasal secretions and endotracheal aspiration fluid between January 2010–March 2011 in a university hospital with more than 1500 beds. This study was conducted in accordance with revised Helsinki Declaration and approved by the institutional clinical research ethic commitee.

All samples were cultured on suitable bacteriological media and identified by the conventional method. *Staphylococcus spp*. isolates were identified by API-Staph commercial identification kit (API Staph System, bioMèrieux, France).

### Minimum inhibitory concentration of antiseptics

MICs of BAC and CHDG were detected by a modified agar dilution method according to the recommendations of Clinical and Laboratory Standards Institute (CLSI) [20]. The MICs of the BAC and CHDG in negative control strain (*S.aureus ATCC 6538*) which is negative for antiseptic resistance genes were considered as baseline and any MICs above these values were accepted as “tolerance concentration”.

### Antibiotic susceptibility test

In this study, antibiotic sensitivity testing was performed via disc diffusion method by using following antibiotics; tetracycline, ciprofloxacin, tobramycin, trimethoprim-sulfamethoxazole, rifampin, and chloramphenicol. Methicillin and iMLSB (inducible macrolide-lincosamide-streptogramin B) resistance tests were implemented to *Staphylocccus spp*. isolates, while vancomycin resistance test to *Enterococcus spp*. isolates. All tests were performed according to CLSI [21] criteria.

The antibiotic susceptibility discs were purchased from OXOID (Hemakim, Istanbul, Turkey).

### Detection of antiseptic resistant genes by multiplex PCR

Total genomic DNA of the strains were extracted using High Pure PCR Template Preparation Kit (Roche, Germany). All DNA extracts were stored at +4 °C prior to PCR.

A single primer pair was used for amplifying both *qacA* and *qacB* due to seven base difference between *qacA* and *qacB* genes. We made two multiplex PCR sets; one included primers of *A/B* and *smr* and second one contained primers of *qacG*, *qacH*, and *qacJ* genes as previously published [15, 22]. All primer sequences are shown in Table 1.

**Table 1:** Sequences of PCR primers

Primer	Gene	Primer Sequence (5′-3′)	Product Size	Reference
*qacA/B F*	*qacA/B*	GCA GAA AGT GCA GAG TTC G	361 bp	Noguchi et al. [22]
*qacA/B R*	*qacA/B*	CCA GTC CAA TCA TGC CTG		
*smr F*	*smr*	GCC ATA AGT ACT GAA GTT ATT GGA	195 bp	Noguchi et al. [22]
*smr R*	*smr*	GAC TAC GGT TGT TAA GAC TAA ACC T		
*qacG F*	*qacG*	CAA CAG AAA TAA TCG GAA CT	275 bp	Bjorland et al. [15]
*qacG R*	*qacG*	TAC ATT TAA GAG CAC TAC A		
*qacH F*	*qacH*	ATA GTC AGT GAA GTA ATA G	295 bp	Bjorland et al. [15]
*qacH R*	*qacH*	AGT GTG ATG ATC CGA ATG T		
*qacJ F*	*qacJ*	CTT ATA TTT AGT AAT AGC G	301 bp	Bjorland et al. [15]
*qacJ R*	*qacJ*	GAT CCA AAA ACG TTA AGA		

PCR Master Mix 2X (Fermentas, Canada) was used for PCR assays. According to recommendation of manufacturer, the final volume of each reaction in the PCR was 25 μL. Each reaction contained 12,5 μL master mix, for each primer; 1 μL (10 pmol) of forward primer, 1 μL (10 pmol) of reverse primer, 2 μL of DNA extract and final volume was 25 μL by adding sterile H_2_O. PCR of *qacA/B* and *smr* genes were performed using an initial denaturation step 96 °C for 3 min, followed by 25 cycles of 95 °C for 20 s, 53 °C for 20 s, 72 °C for 20 s, and a final extension step at 72 °C for 5 min [22]. Whereas the cycling conditions for *qacG, qacH and qacJ* genes were as follows: DNA denaturation at 94 °C for 10 min, 25 cycles of 95 °C for 60 s, 48 °C for 45 s, 72 °C for 60 s, and a final extension step at 72 °C for 10 min [15]. Multiplex PCR products were run in 1% gel containing ethidium bromide and photographed under ultraviolet.

Positive control strains (*qacA/B, smr, qacG, qacH, qacJ*) were provided kindly by Jostein Bjorland from Norwegian School of Veterinary Science. *S.aureus ATCC 6538* was used as negative control strain.

### Statistical analysis

The data analysis was conducted with the statistical package, IBM SPSS Statistics 22 (IBM.

SPSS, Turkey). The normal distribution of variables were checked by the Shapiro–Wilk test. Comparison of quantitive data between two groups were performed by Mann Whitney U test, while Continuity Correction and Fisher’s Exact tests were used for comparison of qualitative data between two groups. *P* value less than 0.05 was considered statistically significant.

## Results

We found that 15/29 (51.7%) *Staphylococcus aureus* and 34/40 (85.0%) CNS isolates, for a total of 49/69 (70.0%) *Staphylococcus spp*. isolates harbored at least one antiseptic resistance genes. Among the 49 strains positive for an antiseptic resistance gene; *qacA/B* genes were the most dominant (28/49; 57.1%) followed by *qacJ* (18/49; 36.7%), *qacG* (16/49; 32.6%), and *smr* (11/49; 22.4%) genes. We also found that 30/49 (61.2%) gene positive *Staphylococcus spp*. isolates had only one gene type, whereas the rest of the isolates (19/49; 38.7%) carried more than one gene type. None of the *Staphylococcus spp*. isolates carried the *qacH* gene. In addition, all *Enterococcus spp*. isolates were negative for any resistance genes (Table 2).

**Table 2:** The presence of antiseptic resistance genes in *Staphyloccus spp.*

RESISTANCE GENES (n:49)	MRSA (n: 10)	MSSA (n:19)	MRCNS (n:27)	MSCNS (n:13)
One gene (n: 30)				
*qacA/B* (n:18)	-	-	14	4
*smr (*n:6)	-	2	2	2
*qacG* (n:3)	2	1	-	-
*qacH* (n:0)	-	-	-	-
*qacJ* (n:3)	-	1	1	1
Two genes (n: 14)				
*qacA/B, smr* (n:3)	-	1	2	-
*qacA/B, qacG* (n:1)	1	-	-	-
*qacA/B, qacJ* (n:3)	-	-	1	2
*qacG,qacJ* (n:7)	3	2	2	-
Three genes (n: 5)				
*qacA/B, qacG, qacJ* (n:3)	-	1	-	2
*smr, qacG, qacJ (*n:2)	1	-	-	1

*MRSA* Methicillin-resistant *Staphylococcus aureus*; *MSSA* Methicillin-susceptible *Staphylococcus aureus*; *MRCNS* Methicillin-resistant coagulase-negative staphylococci; *MSCNS* Methicillin-susceptible coagulase-negative staphylococci

The MICs of BAC ranged from 1 to 16 μg/mL, and the MICs of CHDG ranged from 0.75 to 12 μg/mL in *Staphylococcus spp*. Baseline MIC concentrations of BAC and CHDG in the negative control strain (*S.aureus ATCC 6538*) were 4 and 1.5 μg/mL, respectively. Based on these measurements, we found a significant difference in MIC >4 μg/mL BAC between *Staphylococcus spp*. isolates positive and negative for an antiseptic resistance gene (p: 0.002; *p* < 0.01) but this relationship was not statistically significant for MICs of CHDG (**>**1.5 μg/mL; p: 0.925; *p* > 0.01) (Table 3).

**Table 3:** Comparison between MICs of BAC and CHDG, and presence of antiseptic resistance genes in staphylocci isolates

Antiseptic	MIC	Gene (+) Bacteria (*n* = 49)	Gene (−) Bacteria (*n* = 20)	Z	*p*
		*n* (%)	*n* (%)		
BAC	≤4 μg/mL	23 (46.9%)	18 (90%)	9.209	0.002**
	>4 μg/mL	26 (53.1%)	2 (10%)		
CHDG	≤1.5 μg/mL	32 (65.3%)	14 (70%)	0.009	0.925
	>1.5 μg/mL	17 (34.7%)	6 (30%)		

*Continuity Correction*

**p < 0.01

All *Enterococcus spp*. isolates in this study were negative for antiseptic resistance genes. MIC levels of BAC and CHDG were 8–16 μg/ml and 6–12 μg/mL, respectively. We found that MICs of BAC and CHDG of VRE isolates were significantly higher than those of vancomycin-susceptible enterococci (VSE) isolates (*p* < 0.01) (Table 4).

**Table 4:** Comparison between MIC (μg/mL) of BAC and CHDG in VSE and VRE isolates

MIC (μg/ml)	VSE (*n* = 56)	VRE (*n* = 13)	Z	*p*
	Mean ± SD (Median)	Mean ± SD (Median)		
BAC (8–16 μg/mL)	8.00 ± 0.00 (8)	11.69 ± 4.15 (8)	-5.282	0.001**
CHDG (6–12 μg/mL)	11.78 ± 1.12 (12)	10.15 ± 2.88 (12)	−3.112	0.002**

*Mann Whitney U Test*

**p < 0.01

A comparison of antibiotic resistance and the presence of antiseptic resistance genes in *Staphylococcus spp*. are shown in Table 5.

**Table 5:** Comparison between the antibiotic resistances and presence of antiseptic resistance genes in staphylococci isolates

Antibiotic	Gene (+) Bacteria (*n* = 49)	Gene (−) Bacteria (*n* = 20)	χ^2^	*p*
	*n* (%)	*n* (%)		
iMLSB	22 (44.9%)	10 (50%)	0.014	0.905
Tetracycline	21 (42.9%)	8 (40%)	0.001	1.000
Ciprofloxacin	16 (32.7%)	5 (25%)	0.115	0.735
Tobramycin	14 (28.6%)	5 (25%)	0.001	1.000
SXT	12 (24.5%)	2 (10%)	-	0.322
Rifampin	11 (22.4%)	7 (35%)	0.601	0.438
Chloramphenicol	6 (12.2%)	0 (0%)	-	0.171

*Continuity Correction and Fisher’s Exact Test*

*iMLSB* Inducible macrolide-lincosamide-streptogramin B; *SXT* Trimethoprim-sulfamethoxazole

## Discussion

MRSA, MRCNS, and VRE strains are opportunistic pathogens transmitted by the hands of health care workers. BAC and CHDG are handwashing and skin antiseptics used extensively to the control and prevent hospital infections. Many studies have shown both the presence of plasmid-mediated antiseptic resistance genes such as *qacA/B*, *smr*, *qacG*, *qacH*, and *qacJ* and reduced susceptibility to antiseptic agents. Other researchers have reported that plasmids carry antiseptic resistance genes together with antibiotic resistance genes and contribute to the development of resistance in pathogens [23–25]. In this study, we tried to find a possible association between the presence of antiseptic resistance genes and reduced susceptibility to antiseptics or resistance to various antibiotics in clinical isolates of *Staphylococcus spp*. and *Enterococcus spp*.. We observed that 15 (51.7%) of 29 *S.aureus* and 34 (85.0%) of 40 CNS isolates, for a total of 49 (71%) out of 69 staphylococci isolates harbored at least one antiseptic resistance gene. We also determined that among the 49 *Staphylococcus spp*. isolates positive for a gene, *qacA/B* genes were the most dominant (57.1%) followed by *qacJ* (36.7%), *qacG* (32.6%), and *smr* (22.4%) genes. The *qacH* gene was not found in any tested isolates. Our *Enterococcus spp*. isolates were free from any antiseptic resistance genes.

To date, two studies [26, 27] have been published on the antiseptic resistance genes of staphylococci in Turkey in addition to our previous study in 2012 [25]. Duran et al. [26] reported that the frequency of *qacA/B* and *smr* genes among amikacin-resistant *S.aureus* was 47.4% and 28.9% in Turkey, respectively, and this finding was statistically significant (*p* < 0.05). In the same study, the frequency of *qacA/B* and *smr* in CNS was 37.9% and 20.7%, respectively (*p* < 0.05). In another study conducted in Turkey, Aykan et al. [27] reported that 11.6% of MRSA isolates harbored *qacA/B* resistance genes. In this study, we found that 10% of our MRSA strains harbored the *qacA/B* gene, and our results were compatible with the results of Aykan et al. [27]. In our previous study, (25) we found that *smr* genes were more prevalent (36.0%) in MRSA whereas *qacA/B* genes more prevalent (4.0%) in MSSA strains. In addition, the presence of the iMLSB resistance phenotype in 8/18 (44.5%) *smr*-positive strains compared to 2/32 (6.25%) *smr-*negative strains was statistically significant (*p* < 0.001) [25]. However, unlike our previous work, *smr* and *qacA/B* genes existed in equal frequency (10.0%) in MRSA strains in this study. In addition*, smr* genes were more prevalent (15.7%) than *qacA/B* (10.5%) in MSSA strains. Moreover, we could not find any significant relationship between the presence of antiseptic resistance genes and antibiotic resistance.

Mayer et al. [7] reported results of the SENTRY European study group on the distribution of *qacA/B* and *smr* in 297 MRSA and 200 MSSA strains isolated between 1997 and 1999 in 24 different European university hospitals in 14 countries. They found that 42.0% of *S.aureus* (63.0% of MRSA and 12.0% of MSSA) harbored *qacA/B* genes, and *qacA/B* genes were more prevalent than the *smr* gene, which was detected in 5.8% of *S. aureus* (6.4% of MRSA and 5.0% of MSSA). They emphasized that the prevalence of antiseptic resistance genes is a widespread problem in European hospitals. Our study demonstrated that the presence of *qacA/B* genes in clinical *S.aureus* strains was lower (10.3%) than *smr* genes, which were more prevalent (13.8%) than European strains. Vali et al. [10] reported that in the UK, *smr* (44.2%) genes were most prevalent, followed by *qacA/B* (8.3%) and *qacH* (3.3%), and that *qacA/B* and *smr* were detected concomitantly in 4.2% of isolates; however they did not find *qacG* in 120 clinical MRSA strains. In contrast to Vali et al. we found a high frequency of *qacG* (7/10; 70.0%) in MRSA strains; but the *qacH* gene was not seen. We also detected *qacA/B* genes concomitantly with *smr* in 5.2% (1/19) of MSSA and 7.4% (2/27) of MRCNS, with *qacG* in 50.0% (5/10) of MRSA, and *qacJ* in 11.2% (3/27) of MRCNS and 38.4% (5/13) of MSCNS.

Longtin et al. [28] reported that *smr* genes were more frequent (7.0%) than *qacA/B* genes (2%) in 334 MRSA isolates collected from two Canadian intensive care units between 2005 and 2009, and no strain contained both genes. Noguchi et al. [22] detected *qacA/B* genes in 14.0% and *smr* genes in 28.0% of 71 clinical MRSA isolates in Japan. Alam et al. [29] reported that in 522 clinical *S. aureus* isolates from a hospital in Japan, *qacA/B* was more prevalent in MRSA (32.6%) and more prevalent in MSSA (7.5%) than *smr* genes, which had a frequency of 3.3% in MRSA and 5.9% in MSSA.

Zhang et al. [30] reported that 50.0% of MRSA and 16% of MSSA strains were positive for *qacA/B* gene, and this difference was statistically significant (p: 0.003). In our study, *smr* and *qacA/B* genes existed in equal frequency (10.0%) in MRSA strains, and *smr* genes were more prevalent (15.7%) than *qacA/B* (10.5%) in MSSA strains.

Zhang et al. [30] reported that CNS carried more *qacA/B genes* (56.7%) and *smr* genes (18.1%) than *S. aureus* (41.2% and 11.8%, respectively) in strains isolated from nares of nurses in a hospital in Hong Kong. In the same study, they detected a significant difference in the frequency of *qacA/B* between MRCNS (66.9%) and MSCNS (35.1%) strains (*p* < 0.001). They observed similar findings for *smr* (*p*: 0.001). In our study, the frequency of *qacA/B* and *smr* genes in MRCNS (63% and 14.8%, respectively) was very close to that of MSCNS (61.5% and 23.0%, respectively).

Ye et al. [16] examined the frequency of *qacG*, *qacH* and *qacJ* in 237 *S. aureus* (including 12 MRSA) and 604 CNS isolates (139 of which were methicillin resistant). They found that *S. aureus* isolates from a nurse (1.9%) harbored *qacJ*. Of the *qacG*-positive isolates, one of them harbored *qacA/B* and another harbored *smr*. In the same study, they detected that MICs of gene-positive strains were 2-fold higher than those of negative controls, and the presence of a second *qac* gene further elevated the MICs. The frequency of resistance to gentamicin, fusidic acid, clindamycin, and tetracycline increased in gene-positive isolates, but the MRSA isolates did not harbor these genes.

Liu et al. [11] found that 94.6% (53/56) of QAC-tolerant *S.aureus* isolates was positive for the *qacA/B* gene. The frequencies of *smr* and *qacH* were 3.6% and 7.1%, respectively. *QacG* was not detected in any isolates. The researches concluded that *S. aureus* isolates of China could survive at proper in-use concentrations of some biocides.

Zhang et al. [30] found an association between the presence of antiseptic resistance genes, and resistance to some anibiotics (cefoxitin, penicillin, ciprofloxacin, SXT, tetracycline, clindamycin) and reduced susceptibility to antiseptics (BAC: MIC ≥4 μg/mL, and CHDG: MIC ≥2–4 μg/mL). We also found a significant difference (*p* < 0.01) between the presence of antiseptic resistance genes and the BAC value (MIC > 4 μg/mL) but not antibiotic resistance (*p* > 0.05) in staphylococci isolates.

A limited number of antiseptic resistance gene studies have involved clinical *Enterococcus spp*. isolates. Bischoff et al. [17] found that 1 out of 42 (2.38%) *E. faecalis* isolates from blood samples carried *qacA/B*; 1 out of 109 (0.92%) *E. faecalis* isolates from stool samples harbored *smr*; and no *Enterococcus spp*. isolates were positive for *qacG*, *qacH*, or *qacJ* genes. In contrast to Bischoff et al. [17] we could not find any *qacA/B* and *smr* genes in *Enterococcus spp*. Thus far, no *qac* genes have been reported in enterococci other than *qacC*, *qacEΔ1, qacZ* and *qacA/B*. We also did not find *qacG*, *qacH*, or *qacJ* genes in enterococci isolates.

Bhardaj et al. [31] reported that MIC values of *E. faecium* and *E. faecalis* to CHDG ranged from 0.5 to 16 μg/mL. They also found that VanA-type resistance genes (VRE) are induced by sub-bactericidal levels of CHDG. It is a major concern whether exposure to sub-MIC CHDG results in cross-resistance to antibiotics in clinical use. MICs of BAC and CHDG in our *Enterococci spp*. isolates ranged from 8 to 16 μg/mL and 6 to 12 μg/mL, respectively. However there was a statistically significant difference (*p* < 0.01) of MICs in BAC and CHDG between VRE and VSE strains.

Our study has some limitations. This study focused on antiseptic resistance genes such as *qacA/B*, *smr*, *qacG*, *qacH*, and *qac J*, which are mostly isolated in Gram-positive bacteria, but other genes, such as *qacEΔ, qac F*, and *qacZ*, should be investigated in the future. In addition, the small sample size might have affected the possible relationship between antiseptic resistance genes and the resistance pattern of antibiotics and antiseptic agents.

## Conclusion

The frequency of antiseptic resistance genes was high (71.0%) in our *Staphylococcus spp*. and absent (0%) in *Enterococcus spp*. isolates. The frequency of *qacA/B* and *smr* genes was higher (62.5% and 17.5%, respectively) in CNS isolates compared to *S. aureus* strains (10.3% and 13.8%, respectively). In contrast, the frequency of *qacG* and *qacJ* genes was higher (37.9% and 27.5%, respectively) in *S. aureus* strains compared to CNS strains (12.5% and 25.0%, respectively). We found statistically significant (*p* < 0.01) association between the presence of antiseptic resistance genes and the MIC of BAC (>4 μg/mL) in staphylococci. They may be a marked difference in the frequency and type of antiseptic resistance genes between countries or even between different hospitals in the same country, and therefore each hospital should review its antimicrobial policy and support continuing education to avoid developing new antimicrobial resistance mechanisms.

## Abbreviations

BAC: Benzalkonium chloride
CHDG: Chlorhexidine digluconate
CLSI: Clinical and Laboratory Standards Institute
iMLSB: Inducible macrolide-lincosamide-streptogramin B
MIC: The minimum inhibitory concentration
MRCNS: Methicillin-resistant coagulase-negative staphylococci
MRSA: Methicillin-resistant *Staphylococcus aureus*
MSCNS: Methicillin-susceptible coagulase-negative staphylococci
MSSA: Methicillin-susceptible *Staphylococcus aureus*
SXT: Trimethoprim-sulfamethoxazole
VRE: Vancomycin resistant enterococci
VSE: vancomycin susceptible enterococci
